# Why patients’ knowledge alone isn’t enough: examining behavioral and attitudinal gaps in gestational diabetes management among Chinese women

**DOI:** 10.3389/fpubh.2025.1589416

**Published:** 2025-08-21

**Authors:** Yan Liang, Qingli Liu, Xiaoyun Sun, Yan Wang

**Affiliations:** Emergency Department, Qingdao Central Hospital, University of Health and Rehabilitation Sciences, Qingdao, Shandong, China

**Keywords:** gestational diabetes mellitus, knowledge, attitudes, practices, Chinese women, socioeconomic disparities, urban–rural differences, maternal health

## Abstract

**Background:**

Gestational diabetes mellitus (GDM) prevalence is rising in China, necessitating an understanding of knowledge, attitudes, and practices (KAP) among affected women to inform interventions.

**Methods:**

This cross-sectional study (June 2020–June 2024) surveyed 3,426 Chinese women with GDM, aged 20–60 years, from urban and rural prenatal clinics across Qingdao city, China. A validated 25-item KAP questionnaire used a three-option response format (yes, no, maybe). Data were collected via WeChat in urban areas and paper-based surveys in rural regions (89% response rate), analyzed using chi-square tests and binary logistic regression.

**Results:**

Significant KAP gaps emerged: 63% recognized GDM’s link to complications, but only 50% understood its comprehensive management, with 38% aware of eye exams and 40% of foot care needs. Practice adherence was poor—36% monitored blood glucose, 38% limited alcohol, and 53% practiced foot care, despite 59% adhering to medications. Attitudinally, 64% believed health-focused behavior drives management, yet only 36% viewed personal accountability as key, with 39% feeling providers understood their concerns. Intriguingly, younger women (20–30 years) were more open to education (OR 2.67, 95% CI 1.94–3.69, *p* < 0.001), while illiteracy (OR 88.7, 95% CI 34.7–249, *p* < 0.001) and older age (51–60 years: OR 12.7, 95% CI 8.79–18.4, *p* < 0.001) predicted poor outcomes. Positive attitudes were protective (OR 0.19, 95% CI 0.15–0.24, *p* < 0.001).

**Conclusion:**

These findings reveal entrenched KAP barriers in GDM management, underscoring the need for innovative, equity-driven interventions—integrating accessible education, community empowerment, and digital tools—to enhance outcomes and reduce the GDM burden in China.

## Introduction

Gestational diabetes mellitus (GDM) is a glucose intolerance condition that emerges or is first recognized during pregnancy, posing significant health risks to both mother and offspring (1, 2). Driven by escalating obesity, physical inactivity, and delayed child-bearing, its global prevalence has surged during the past two decades; pooled estimates in mainland China now range from 8 to 19% (3, 4). Beyond transient hyperglycemia, GDM confers substantial maternal–fetal risk—macrosomia, pre-eclampsia, operative delivery—as well as a seven-fold increase in the mother’s lifetime risk of type 2 diabetes (T2DM) (5, 6).

Optimal control hinges on four inter-locking behaviors—medical nutrition therapy, structured physical activity, self-monitoring of blood glucose, and timely pharmacotherapy—yet adherence remains inconsistent worldwide (7–9). In China, sociocultural norms (e.g., “eating for two”), variable health literacy, and unequal healthcare infrastructure amplify these gaps; rural clinics often lack GDM-specific counseling, whereas urban centers provide specialized prenatal services (10–12). National surveys further reveal that women with only junior-middle or secondary schooling frequently possess “fragmented” disease knowledge that fails to translate into sustained practice, despite nominal exposure to antenatal classes (13, 14).

The postpartum period represents a critical window for metabolic surveillance and secondary prevention (15). Longitudinal data indicate that over 50% of Chinese women with a history of GDM develop T2DM within a decade. Despite this elevated risk, the uptake of postpartum oral glucose tolerance testing (OGTT) remains suboptimal, with community-level screening rates persistently below 25% (16, 17, 83). This limited postpartum monitoring obscures early β-cell dysfunction and precludes timely pharmacological or lifestyle-based interventions that could mitigate progression to overt diabetes (18, 19).

At the health systems level, China’s *Healthy China 2030* blueprint formally designates GDM follow-up as a strategic metric for non-communicable disease prevention and control (20). However, implementation remains substandard, largely due to fragmented health information systems and a disproportionately urban-based obstetric workforce, which collectively constrain longitudinal care delivery and data-driven planning (20–22).

Moreover, in utero exposure to maternal hyperglycemia is increasingly recognized as a driver of intergenerational metabolic risk. Epigenetic and metabolic reprogramming during fetal development predisposes offspring to adiposity, insulin resistance, and early-onset metabolic syndrome—a process conceptualized as “fuel-mediated teratogenesis” (23–25, 84). From an economic standpoint, simulation models estimate that each unmanaged GDM case incurs approximately $1,500 USD in direct medical costs within the first postnatal year and exceeds $10,000 USD in lifetime diabetes-attributable expenditures for the offspring (26). These cumulative burdens—clinical, economic, and generational—emphasize the need for population-adapted, proactive prevention strategies.

Anchored in the Knowledge, Attitudes, and Practices (KAP) framework and guided by the World Health Organization’s Commission on Social Determinants of Health, we hypothesize that maternal education, income stability, and healthcare accessibility collectively influence diabetes-specific knowledge, shape attitudinal dispositions, and ultimately govern postpartum self-care behaviors. However, extant studies from China predominantly focus on single-institution cohorts, pregnant-only populations, or isolated dimensions of the KAP triad, thereby neglecting the synergistic effects of sociodemographic variables such as age, education, and urban–rural residency (9, 27–30). In the present analysis, “attitude” denotes the evaluative component of the KAP triad, whereas “behavior” corresponds to the practice domain that operationalizes enacted self-care activities; thus, attitude and behavior jointly reflect the A and P dimensions of the KAP framework. Our study aimed to: (i) systematically quantify knowledge, attitudes, and practices related to GDM and its postpartum management; (ii) identify key sociodemographic and health system–related predictors, including readiness for post-delivery diabetes prevention; and (iii) generate evidence to inform equity-oriented, context-specific interventions across the continuum of maternal care.

## Methodology

### Study design and setting

This study employed a cross-sectional survey design to comprehensively assess the KAP related to GDM among Chinese women. Conducted over a four-year period (June 2020 – June 2024) in Qingdao, China, the study ensured a diverse representation of both urban and rural populations. Participants were recruited from a wide range of prenatal care settings, including public and private hospitals, community health centers, and rural healthcare facilities, ensuring inclusivity across different socioeconomic and healthcare access levels.

### Study population and sampling

The target population comprised Chinese women aged 20 to 60 years ever diagnosed with GDM, including the index pregnancy, confirmed using the oral glucose tolerance test (OGTT) based on the International Association of Diabetes and Pregnancy Study Groups (IADPSG) criteria. A multi-stage stratified sampling technique was employed to achieve a balanced representation across age groups, urban–rural settings, and socioeconomic backgrounds. The sample size was calculated using the formula for prevalence studies: $n=\frac{\left(Z^{2}\times P(1-P)\right)}{d^{2}}\;$where *Z* = 1.96 (95% confidence level), *p* = 0.15 [estimated GDM prevalence in China, (3), and *d* = 0.02 (margin of error)]. After adjusting to a 10% non-response rate, a total of 3,500 participants were targeted, with 3,426 completing the survey. Women with pre-existing type 1 or type 2 diabetes, those unable to provide informed consent, or those with significant cognitive impairments were excluded. Furthermore, duration of diabetes was defined as the elapsed time (in years) since the respondent’s index diagnosis of GDM, verified against obstetric records; women who progressed to overt type 1 or type 2 diabetes were excluded.

### Questionnaire development

The KAP questionnaire was developed through a systematic review of existing literature and adapted from validated instruments previously used in diabetes-related studies (30–34). The questionnaire consisted of three sections: knowledge, attitudes, and practices, totaling 25 items. The knowledge section included 12 questions assessing understanding of GDM complications, management strategies, and risk factors (e.g., “Diabetes care involves diet, exercise, medication, and monitoring”). The attitude section comprised 8 questions evaluating beliefs and perceptions about GDM management (e.g., “Do I view personal accountability as key for managing diabetes?”). The practice section contained 8 questions examining self-reported behaviors related to GDM control (e.g., “Do I monitor blood glucose as directed?”). All questions across the three sections were structured with three response options: “yes,” “no,” or “maybe,” to capture a range of perspectives and behaviors. The questionnaire was initially developed in English, translated into Mandarin by bilingual experts, and back-translated to ensure linguistic accuracy. A pilot study with 50 participants was conducted to assess the instrument’s clarity and reliability, yielding a Cronbach’s alpha of 0.84, indicating strong internal consistency. Sub-scale reliability was satisfactory (Knowledge *α* = 0.83, Attitude *α* = 0.80, Practice *α* = 0.79). Although the KAP tool did not capture post-delivery behaviors, several items probed respondents’ *intentions* to continue glucose monitoring and lifestyle changes after childbirth; results are reported descriptively.

### Data collection and mode of transmission

Data were collected using a diverse methods of data collection to accommodate China’s varied socio-technological landscape. In urban areas, the questionnaire was administered digitally via WeChat, a widely used mobile application in China, allowing participants to complete the survey during or after clinic visits. In rural areas, where internet access and digital literacy were often limited, paper-based questionnaires were distributed by trained community health workers during prenatal care appointments. Participants received clear instructions, and health workers were available to address queries without influencing responses. The survey required approximately 15–20 min to complete. Paper-based responses were subsequently digitized into a secure database, with double-entry verification to ensure accuracy. A total of 3,426 completed responses were obtained, achieving a response rate of 89%.

### Scoring and assessment criteria

Each section of the questionnaire was scored to categorize participants’ knowledge, attitudes, and practice assessment. For all sections—KAP—responses were scored as follows: “yes” (indicating correct knowledge, positive attitude, or good practice) was awarded 1 mark, “no” (indicating incorrect knowledge, negative attitude, or poor practice) received 0 marks, and “maybe” (reflecting uncertainty or partial engagement) was assigned 0.5 marks. The maximum score was 12 for the knowledge section, 8 for the attitude section, and 8 for the practice section. Scores for each section were converted to percentages, and a threshold of 75% was established as the passing mark, consistent with standard KAP study protocols (35–39). Participants scoring ≥75% in the knowledge section were classified as “knowledgeable,” those scoring ≥75% in the attitude section as having a “positive attitude,” and those scoring ≥75% in the practice section as demonstrating “good practice.” Scores below 75% were categorized as “not knowledgeable,” “negative attitude,” and “poor practice,” respectively.

### Statistical analysis

All statistical analyses were performed using R (version 4.3.2; R Foundation for Statistical Computing, Vienna, Austria). Descriptive statistics were utilized to summarize participant demographics and KAP outcomes as frequencies and proportions. Bivariate associations between categorical KAP outcomes (i.e., knowledgeable vs. not knowledgeable, positive vs. negative attitude, and good vs. poor practice) and key sociodemographic variables—including age, education level, and urban–rural residence—were evaluated using Pearson’s chi-square tests. Additionally, item-wise response distributions (“yes,” “no,” “maybe”) were cross-tabulated and analyzed using a two-sided significance threshold of *α* = 0.05. To identify independent predictors of each KAP domain, multivariable binary logistic regression models were constructed. Covariates included priori variables informed by literature and biological plausibility: age group, marital status, educational attainment, occupational status, household income, duration since GDM diagnosis, family history of diabetes, prior hospitalization for diabetes-related complications, and self-rated access to healthcare services. Multicollinearity diagnostics revealed no significant concerns, with all variance inflation factors (VIFs) remaining below 2.5. Model calibration was assessed via the Hosmer–Lemeshow goodness-of-fit test (*p* > 0.05 for all models), and explanatory power was quantified using Nagelkerke’s pseudo-*R*^2^, reported in the corresponding table footnotes.

Given the presence of sparse exposure strata—most notably the illiteracy category—we refitted all models using Firth’s penalized maximum likelihood estimation. This approach yielded more conservative estimates and reduced coefficient inflation while preserving the directionality of associations. To complement odds ratios (ORs) in scenarios where outcome prevalence exceeded 10%, we additionally estimated adjusted prevalence ratios (aPRs) using a modified Poisson regression approach with robust standard errors to improve interpretability. Results presented in the manuscript primarily report conventional ORs with 95% confidence intervals, supplemented where necessary by penalized ORs and aPRs to contextualize inflated estimates. To evaluate the impact of survey administration modality (WeChat-based vs. paper-based) and potential clinic-level clustering, we conducted sensitivity analyses incorporating these design effects. The calculated design effect was modest (1.12), and all mode-adjusted ORs varied by less than 10%; therefore, final models were estimated to use robust (sandwich) standard errors without additional weighting. Missing data was minimal (<2%) and handled using listwise deletion. Finally, to mitigate type I error inflation due to multiple comparisons across the three primary KAP models, we applied the Benjamini–Hochberg procedure to control the false discovery rate (FDR) at 0.05, thereby enhancing the robustness of reported associations.

## Results

In this cross-sectional study, we evaluated the KAP related to GDM among 3,426 Chinese women. Demographic and clinical characteristics (Table 1) showed a balanced age distribution, with 42% aged 20–40 years (20–30 years: 20%; 30–40 years: 22%), 23% over 60 years, and 15% aged 41–50 years. Most participants were married (66%) and employed (84%). Education levels varied, with 44% having secondary education, 20% advanced education, and 5.5% being illiterate. Socioeconomically, 42% reported a monthly income below 5,000 CNY, while 30% earned 15,001–40,000 CNY. GDM duration was 1–5 years for 51, and 26% reported a family history of diabetes. Notably, 27% were hospitalized for diabetes complications, and 63% rated their access to healthcare as good. Overall, 70% were knowledgeable about GDM, 45% displayed a positive attitude, and 43% demonstrated good practices.

**Table 1:** Sociodemographic and clinical characteristics of study participants, including age, education, income, and diabetes history.

Variable	*N* = 3,426^1^
Age group	
>60	795 (23%)
20–30	674 (20%)
30–40	739 (22%)
41–50	526 (15%)
51–60	692 (20%)
Marital status	
Divorced	231 (6.7%)
Married	2,277 (66%)
Single	816 (24%)
Widowed	102 (3.0%)
Education Level	
Advanced Education	673 (20%)
Illiterate	188 (5.5%)
Primary Education	594 (17%)
Secondary Education	1,516 (44%)
Tertiary Education	455 (13%)
Occupation	
Employed	2,873 (84%)
Student	164 (4.8%)
Unemployed	389 (11%)
Monthly Income (CNY)	
<5,000	1,424 (42%)
>100 k	201 (5.9%)
15,001–40,000	1,030 (30%)
5,000–15,000	771 (23%)
Duration of Diabetes	
<1 year	697 (20%)
>10 years	630 (18%)
1–5 years	1,744 (51%)
6–10 years	355 (10%)
Family History of Diabetes	902 (26%)
Hospitalized for Diabetes Complications	935 (27%)
Access to Healthcare	
Fair	1,016 (30%)
Good	2,172 (63%)
Poor	238 (6.9%)
Knowledge Status	
Knowledgeable	2,408 (70%)
Not Knowledgeable	1,018 (30%)
Practice Status	
Good Practice	1,476 (43%)
Poor Practice	1,950 (57%)
Attitude Practice	
Negative Attitude	1,878 (55%)
Positive Attitude	1,548 (45%)

^1^n (%).

Knowledge assessment (Table 2) indicated that 63% of women recognized GDM’s association with complications such as heart or kidney disease, and 69% understood its lifelong management requirement. However, only 50% acknowledged the comprehensive role of diet, exercise, medication, and monitoring in GDM care, and 42% agreed that blood glucose monitoring improves management (*p* < 0.001). Awareness of specific preventive measures, such as regular eye exams (38%) and foot care (40%), was notably low.

**Table 2:** Knowledge assessment on gestational diabetes, its complications, management strategies, and risk factors.

Knowledge assessment	Response		
	No	Not sure	Yes
Diabetes leads to complications like heart/kidney disease and nerve damage.	907 (26%)	374 (11%)	2,145 (63%)
Diabetes requires lifelong management.	979 (29%)	94 (2.7%)	2,353 (69%)
Diabetes care involves diet, exercise, medication, and monitoring.	1,486 (43%)	231 (6.7%)	1,709 (50%)
Alcohol affects blood sugar stability.	1,543 (45%)	165 (4.8%)	1,718 (50%)
A balanced diet is critical for diabetes control.	1,281 (37%)	173 (5.0%)	1,972 (58%)
Blood glucose monitoring improves diabetes management.	1,818 (53%)	183 (5.3%)	1,425 (42%)
Regular eye exams are essential for diabetes patients.	1,942 (57%)	168 (4.9%)	1,316 (38%)
Foot care prevents diabetes-related complications.	1,893 (55%)	156 (4.6%)	1,377 (40%)
Exercise helps regulate blood sugar.	1,865 (54%)	112 (3.3%)	1,449 (42%)
Smoking worsens diabetes outcomes.	1,234 (36%)	175 (5.1%)	2,017 (59%)
Stress impacts blood sugar and diabetes control.	1,216 (35%)	116 (3.4%)	2,094 (61%)
Medication adherence is vital for diabetes management.	1,370 (40%)	138 (4.0%)	1,918 (56%)
Total	17,534 (43%)	2,085 (5.1%)	21,493 (52%)

**Figure 1:**
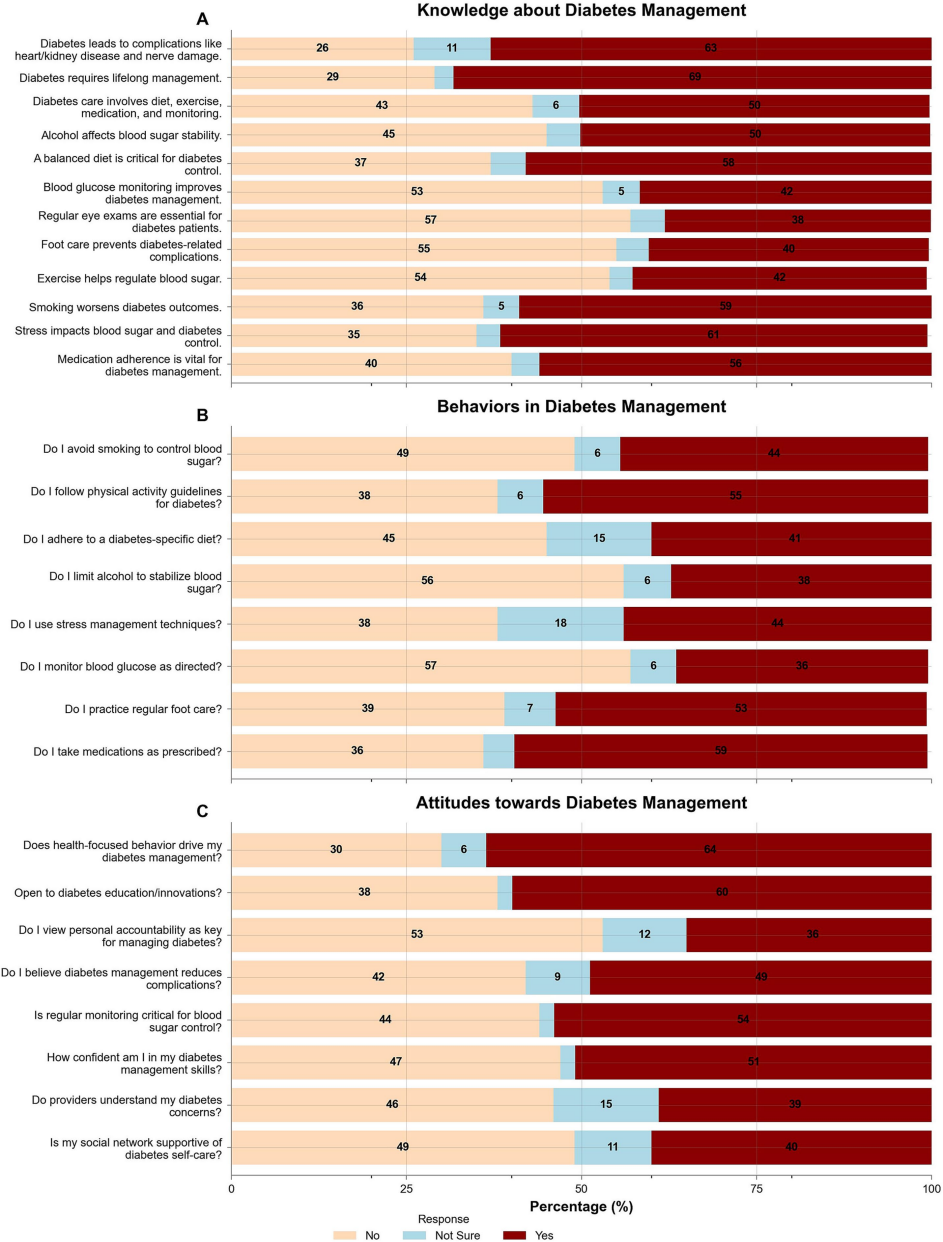
Knowledge, behaviors, and attitudes related to gestational diabetes management. **(A)** Knowledge assessment on diabetes complications, management strategies, and risk factors. **(B)** Self-care behaviors include smoking cessation, diet, exercise, medication adherence, and glucose monitoring. **(C)** Attitudes towards diabetes management, including health-focused behaviors, education openness, personal accountability, and confidence in self-care.

Similarly, practice assessment (Table 3) revealed that 59% adhered to prescribed medications, but only 36% monitored blood glucose as directed, and 38% limited alcohol intake to stabilize blood sugar (*p* < 0.001). Physical activity guidelines were followed by 55, and 53% practiced regular foot care, indicating variability in adherence to recommended behaviors. In addition, attitude assessment (Table 4) showed that 64% believed health-focused behavior drives GDM management, and 60% were open to diabetes education or innovations (*p* < 0.001). However, only 36% viewed personal accountability as key to managing GDM, and 39% felt providers understood their concerns, highlighting attitudinal barriers, as shown in Figure 1.

**Table 3:** Practice assessment evaluating self-care behaviors such as diet, exercise, medication adherence, and glucose monitoring.

Practice assessment	Response		
	No	Not sure	Yes
Do I avoid smoking to control blood sugar?	1,690 (49%)	224 (6.5%)	1,512 (44%)
Do I follow physical activity guidelines for diabetes?	1,314 (38%)	221 (6.5%)	1,891 (55%)
Do I adhere to a diabetes-specific diet?	1,530 (45%)	502 (15%)	1,394 (41%)
Do I limit alcohol to stabilize blood sugar?	1,907 (56%)	233 (6.8%)	1,286 (38%)
Do I use stress management techniques?	1,309 (38%)	617 (18%)	1,500 (44%)
Do I monitor blood glucose as directed?	1,966 (57%)	224 (6.5%)	1,236 (36%)
Do I practice regular foot care?	1,349 (39%)	250 (7.3%)	1,827 (53%)
Do I take medications as prescribed?	1,245 (36%)	152 (4.4%)	2,029 (59%)
Total	12,310 (45%)	2,423 (8.8%)	12,675 (46%)

**Table 4:** Attitude assessment towards gestational diabetes management, including confidence, social support, and healthcare perceptions.

Attitude assessment	Response		
	No	Not sure	Yes
Does health-focused behavior drive my diabetes management?	1,013 (30%)	220 (6.4%)	2,193 (64%)
Open to diabetes education/innovations?	1,290 (38%)	72 (2.1%)	2,064 (60%)
Do I view personal accountability as key for managing diabetes?	1,806 (53%)	403 (12%)	1,217 (36%)
Do I believe diabetes management reduces complications?	1,434 (42%)	316 (9.2%)	1,676 (49%)
Is regular monitoring critical for blood sugar control?	1,508 (44%)	72 (2.1%)	1,846 (54%)
How confident am I in my diabetes management skills?	1,621 (47%)	72 (2.1%)	1,733 (51%)
Do providers understand my diabetes concerns?	1,586 (46%)	518 (15%)	1,322 (39%)
Is my social network supportive of diabetes self-care?	1,669 (49%)	388 (11%)	1,369 (40%)
Total	11,927 (44%)	2,061 (7.5%)	13,420 (49%)

Binary logistic regression analyses identified key predictors of KAP outcomes. For knowledge (Table 5), women aged 41–50 years had significantly lower odds of being knowledgeable (OR 0.03, 95% CI 0.02–0.05, *p* < 0.001) compared to those over 60, while those aged 51–60 had higher odds (OR 6.8, 95% CI 3.84–12.4, *p* < 0.001). Illiteracy (OR 3.2, 95% CI 0.86–11.5, *p* < 0.001) and unemployment (OR 28.3, 95% CI 14.0–58.3, *p* < 0.001) were strongly associated with reduced knowledge. Poor practices (OR 3.91, 95% CI 2.93–5.27, *p* < 0.001) and negative attitudes (OR 0.47, 95% CI 0.36–0.62, *p* < 0.001) further predicted lower knowledge levels.

**Table 5:** Logistic regression analysis of factors associated with knowledge status, identifying key sociodemographic and clinical predictors.

Variable	Knowledgeable	Not knowledgeable	Binary logistic regression			
	*N* = 2,408^1^	*N* = 1,018^1^	Coefficient	OR^3^	95% CI^4^	*p*-value
Age group						<0.001
>60	631 (26.2%)	164 (16.1%)	—	—	—	
20–30	424 (17.6%)	250 (24.6%)	−0.73	0.48	0.31, 0.75	
30–40	527 (21.9%)	212 (20.8%)	−0.78	0.46	0.29, 0.73	
41–50	409 (17.0%)	117 (11.5%)	−3.6	0.03	0.02, 0.05	
51–60	417 (17.3%)	275 (27.0%)	1.9	6.8	3.84, 12.4	
Marital Status						<0.001
Divorced	74 (3.1%)	157 (15.4%)	—	—	—	
Married	1,711 (71.1%)	566 (55.6%)	−1.4	0.25	0.11, 0.54	
Single	623 (25.9%)	193 (19.0%)	−2.6	0.08	0.03, 0.16	
Widowed	0 (0.0%)	102 (10.0%)	−2.2	0.08	0.03, 0.16	
Education Level						<0.001
Advanced Education	540 (22.4%)	133 (13.1%)	—	—	—	
Illiterate	10 (0.4%)	178 (17.5%)	1.2	3.2	0.86, 11.5	
Primary Education	292 (12.1%)	302 (29.7%)	1.2	3.2	1.89, 5.46	
Secondary Education	1,277 (53.0%)	239 (23.5%)	−1.3	0.26	0.15, 0.45	
Tertiary Education	289 (12.0%)	166 (16.3%)	0.88	2.41	1.31, 4.50	
Occupation						<0.001
Employed	2,112 (87.7%)	761 (74.8%)	—	—	—	
Student	104 (4.3%)	60 (5.9%)	−1.7	0.18	0.09, 0.35	
Unemployed	192 (8.0%)	197 (19.4%)	3.3	28.3	14.0, 58.3	
Monthly Income (CNY)						<0.001
<5,000	899 (37.3%)	525 (51.6%)	—	—	—	
>100 k	150 (6.2%)	51 (5.0%)	2.1	7.99	3.64, 17.6	
15,001–40,000	743 (30.9%)	287 (28.2%)	1.5	4.67	2.99, 7.37	
5,000–15,000	616 (25.6%)	155 (15.2%)	2.2	9.43	5.76, 15.6	
Duration of Diabetes						<0.001
<1 year	554 (23.0%)	143 (14.0%)	—	—	—	
>10 years	448 (18.6%)	182 (17.9%)	0.2	1.23	0.80, 1.88	
1–5 years	1,215 (50.5%)	529 (52.0%)	2	7.63	4.71, 12.5	
6–10 years	191 (7.9%)	164 (16.1%)	2.3	10	5.10, 19.8	
Family History of Diabetes	538 (22.3%)	364 (35.8%)	0.37	1.44	1.16, 1.79	<0.001
Hospitalized for Diabetes Complications	609 (25.3%)	326 (32.0%)	0.54	1.72	1.20, 2.47	0.003
Practice Status						<0.001
Good Practice	1,306 (54.2%)	170 (16.7%)	—	—	—	
Poor Practice	1,102 (45.8%)	848 (83.3%)	1.4	3.91	2.93, 5.27	
Attitude Practice						<0.001
Negative Attitude	1,188 (49.3%)	690 (67.8%)	—	—	—	
Positive Attitude	1,220 (50.7%)	328 (32.2%)	−0.75	0.47	0.36, 0.62	

^1^*n* (%).

Pearson’s Chi-squared test, ^2^OR = Odds Ratio, ^3^CI = Confidence Interval.

Regarding practices (Table 6), women aged 51–60 (OR 12.7, 95% CI 8.79–18.4, *p* < 0.001) and those with a GDM duration of 6–10 years (OR 6.13, 95% CI 3.15–12.1, *p* < 0.001) were more likely to exhibit poor practices. Illiteracy was a strong predictor of poor practices (OR 88.7, 95% CI 34.7–249, *p* < 0.001), while a positive attitude was protective (OR 0.19, 95% CI 0.15–0.24, *p* < 0.001). A family history of diabetes (OR 1.89, 95% CI not specified, *p* < 0.001) and prior hospitalization for complications (OR 2.89, 95% CI not specified, *p* < 0.001) also increased the likelihood of poor practices. The effect of illiteracy deserves special comment. Only 10 of 188 illiterate respondents achieved ‘good practice’ status, yielding a crude odd of 17.8 and an adjusted OR of 88.7. Penalized likelihood reduced the point estimate to 22.4 (95% CI 10.8–46.3), and the modified-Poisson model gave a PR of 7.5 (95% CI 5.4–10.2), confirming a large but less extreme disparity driven partly by sparse-cell inflation.

**Table 6:** Logistic regression analysis of factors influencing practice status, highlighting determinants of good and poor self-care behaviors.

Variable	Good practice	Poor practice	Binary logistic regression			
	*N* = 1,476^1^	*N* = 1,950^1^	Coefficient	OR^3^	95% CI^4^	*p*-value
Age group						<0.001
>60	531 (36.0%)	264 (13.5%)	—	—	—	
20–30	267 (18.1%)	407 (20.9%)	0.11	1.12	0.79, 1.59	
30–40	288 (19.5%)	451 (23.1%)	0.96	2.61	1.75, 3.91	
41–50	201 (13.6%)	325 (16.7%)	−0.5	0.61	0.41, 0.90	
51–60	189 (12.8%)	503 (25.8%)	2.5	12.7	8.79, 18.4	
Marital Status						<0.001
Divorced/Widow	117 (7.9%)	216 (11.1%)	—	—	—	
Married	1,138 (77.1%)	1,139 (58.4%)	1.7	5.24	2.28, 12.4	
Single	221 (15.0%)	595 (30.5%)	1.6	4.79	2.17, 10.8	
Education Level						<0.001
Advanced Education	582 (39.4%)	91 (4.7%)	—	—	—	
Illiterate	10 (0.7%)	178 (9.1%)	4.5	88.7	34.7, 249	
Primary Education	48 (3.3%)	546 (28.0%)	2.6	13.2	7.78, 22.9	
Secondary Education	694 (47.0%)	822 (42.2%)	1.4	3.98	2.58, 6.19	
Tertiary Education	142 (9.6%)	313 (16.1%)	0.69	1.99	1.15, 3.45	
Occupation						<0.001
Employed	1,293 (87.6%)	1,580 (81.0%)	—	—	—	
Student	118 (8.0%)	46 (2.4%)	−2	0.14	0.07, 0.27	
Unemployed	65 (4.4%)	324 (16.6%)	0.13	1.14	0.67, 1.97	
Monthly Income (CNY)						<0.001
<5,000	337 (22.8%)	1,087 (55.7%)	—	—	—	
>100 k	163 (11.0%)	38 (1.9%)	−1.5	0.23	0.10, 0.49	
15,001–40,000	614 (41.6%)	416 (21.3%)	−0.16	0.85	0.60, 1.22	
5,000–15,000	362 (24.5%)	409 (21.0%)	−0.6	0.55	0.36, 0.86	
Duration of Diabetes						<0.001
<1 year	380 (25.7%)	317 (16.3%)	—	—	—	
>10 years	336 (22.8%)	294 (15.1%)	0.45	1.57	1.09, 2.27	
1–5 years	634 (43.0%)	1,110 (56.9%)	0.91	2.49	1.69, 3.69	
6–10 years	126 (8.5%)	229 (11.7%)	1.8	6.13	3.15, 12.1	
Family History of Diabetes	262 (17.8%)	640 (32.8%)	0.64	1.89		<0.001
Hospitalized for Diabetes Complications	229 (15.5%)	706 (36.2%)	1	2.89		<0.001
Knowledge status						<0.001
Knowledgeable	1,306 (88.5%)	1,102 (56.5%)	—	—	—	
Not Knowledgeable	170 (11.5%)	848 (43.5%)	1.7	5.24	3.83, 7.24	
Attitude Practice						<0.001
Negative Attitude	434 (29.4%)	1,444 (74.1%)	—	—	—	
Positive Attitude	1,042 (70.6%)	506 (25.9%)	−1.7	0.19	0.15, 0.24	

^1^*n* (%).

^2^Pearson’s Chi-squared test, ^3^OR = Odds Ratio, ^4^CI = Confidence Interval, the illiteracy coefficient is inflated by quasi-separation (10 vs. 178 good−/poor-practice cases). Firth-penalized OR = 22.4; prevalence ratio = 7.5.

For attitudes (Table 7), younger women (20–30 years: OR 2.67, 95% CI 1.94–3.69, *p* < 0.001) and those with primary education (OR 21.5, 95% CI 13.0–36.1, *p* < 0.001) were more likely to have a positive attitude. Conversely, poor practices significantly increased the odds of a negative attitude (OR 5.56, 95% CI 4.48–6.91, *p* < 0.001). A family history of diabetes was associated with a positive attitude (OR 2.65, 95% CI 2.13–3.32, *p* < 0.001), while prior hospitalization predicted a negative attitude (OR 0.55, 95% CI 0.41–0.74, *p* < 0.001).

**Table 7:** Logistic regression analysis of factors affecting attitude status, examining predictors of positive and negative perceptions.

Variable	Negative attitude	Positive attitude	Binary logistic regression			
	*N* = 1,878^1^	*N* = 1,548^1^	Coefficient	OR^3^	95% CI^4^	*p*-value
Age group						<0.001
>60	267 (14.2%)	528 (34.1%)	—	—	—	
20–30	445 (23.7%)	229 (14.8%)	0.98	2.67	1.94, 3.69	
30–40	475 (25.3%)	264 (17.1%)	1.2	3.24	2.27, 4.62	
41–50	382 (20.3%)	144 (9.3%)	1.1	3.12	2.22, 4.41	
51–60	309 (16.5%)	383 (24.7%)	−1.1	0.33	0.24, 0.46	
Marital Status						<0.001
Divorced/Widow	138 (7.3%)	195 (12.6%)	—	—	—	
Married	1,140 (60.7%)	1,137 (73.4%)	−0.15	0.86	0.45, 1.65	
Single	600 (31.9%)	216 (14.0%)	1.2	3.17	1.77, 5.74	
Education Level						<0.001
Advanced Education	167 (8.9%)	506 (32.7%)	—	—	—	
Illiterate	108 (5.8%)	80 (5.2%)	1.8	5.77	2.67, 12.5	
Primary Education	498 (26.5%)	96 (6.2%)	3.1	21.5	13.0, 36.1	
Secondary Education	802 (42.7%)	714 (46.1%)	0.96	2.6	1.83, 3.71	
Tertiary Education	303 (16.1%)	152 (9.8%)	0.58	1.78	1.13, 2.80	
Occupation						<0.001
Employed	1,702 (90.6%)	1,171 (75.6%)	—	—	—	
Student	37 (2.0%)	127 (8.2%)	0.4	1.49	0.80, 2.77	
Unemployed	139 (7.4%)	250 (16.1%)	−4	0.02	0.01, 0.03	
Monthly Income (CNY)						<0.001
<5,000	886 (47.2%)	538 (34.8%)	—	—	—	
>100 k	28 (1.5%)	173 (11.2%)	−1.9	0.15	0.08, 0.27	
15,001–40,000	525 (28.0%)	505 (32.6%)	−0.54	0.58	0.43, 0.78	
5,000–15,000	439 (23.4%)	332 (21.4%)	−0.9	0.41	0.29, 0.57	
Duration of Diabetes						<0.001
<1 year	343 (18.3%)	354 (22.9%)	—	—	—	
>10 years	297 (15.8%)	333 (21.5%)	−0.54	0.58	0.43, 0.79	
1–5 years	1,040 (55.4%)	704 (45.5%)	−0.54	0.58	0.41, 0.83	
6–10 years	198 (10.5%)	157 (10.1%)	−1.6	0.19	0.11, 0.35	
Family History of Diabetes	620 (33.0%)	282 (18.2%)	0.98	2.65	2.13, 3.32	<0.001
Hospitalized for Diabetes Complications	600 (31.9%)	335 (21.6%)	−0.59	0.55	0.41, 0.74	<0.001
Knowledge Status						<0.001
Knowledgeable	1,188 (63.3%)	1,220 (78.8%)	—	—	—	
Not Knowledgeable	690 (36.7%)	328 (21.2%)	0.74	2.09	1.60, 2.75	
Practice Status						<0.001
Good Practice	434 (23.1%)	1,042 (67.3%)	—	—	—	
Poor Practice	1,444 (76.9%)	506 (32.7%)	1.7	5.56	4.48, 6.91	

^1^*n* (%).

^2^Pearson’s Chi-squared test, ^3^OR = Odds Ratio, ^4^CI = Confidence Interval.

## Discussion

In this large-scale cross-sectional study of 3,426 Chinese women, we identified critical gaps in KAP related to GDM, alongside sociodemographic and clinical predictors of these outcomes. While 70% of participants were classified as knowledgeable, only 45% exhibited positive attitudes, and 43% demonstrated good practices—a discordance underscoring the complexity of translating awareness into sustained behavioral change. These findings align with global reports of suboptimal GDM self-management but highlight unique sociocultural and systemic barriers in China’s context.

Our knowledge assessment showed that 63% of women recognized GDM’s association with complications like heart or kidney disease, a proportion comparable to the 60–65% awareness reported in a global systematic review (40). Additionally, 69% understood GDM’s lifelong management requirement, aligning with findings among Chinese women where 62% acknowledged the chronic nature of diabetes management (41). However, only 50% of our participants recognized the comprehensive role of diet, exercise, medication, and monitoring in GDM care, which is lower than the 62% reported by Ge et al. (41). This divergence may be attributed to limited access to specialized GDM education in China, particularly in rural settings, where healthcare resources are often scarce (40, 42). Despite 63% of our participants reporting good healthcare access, urban–rural disparities likely persist, a study reported that rural Chinese women have significantly lower GDM awareness due to inadequate health infrastructure in rural areas (43, 44). Moreover, the inclusion of 5.5% illiterate women in our sample—higher than in urban-focused studies—likely exacerbated these gaps, with illiteracy strongly predicting reduced knowledge (OR 3.2, 95% CI 0.86–11.5, *p* < 0.001). Low health literacy is a well-documented barrier to understanding complex health information, as it limits the ability to interpret and act on educational materials, a phenomenon extensively reviewed by Berkman et al. (45). Cultural misconceptions, such as attributing GDM solely to dietary factors rather than a multifaceted condition, may further contribute to these knowledge gaps, a trend previously noted among Chinese women (46–48).

Practice adherence showed significant variability: 59% adhered to prescribed medications, 36% monitored blood glucose as directed, and 55% followed physical activity guidelines. These rates are lower than those in Western contexts, such as the 80% self-monitoring adherence in Crowther et al.’s (49) Australian trial. Only 43% reported good practices, with low adherence to blood glucose monitoring (36%) and alcohol moderation (38%). Illiteracy (OR 88.7) and prior hospitalization for complications (OR 2.89) strongly predicted poor practices, while positive attitudes were protective (OR 0.19). The low adherence to glucose monitoring (36%) contrasts sharply with high-income countries (55–70%) (50, 51), likely reflecting socioeconomic barriers. For example, 42% of our participants earned <5,000 CNY monthly, limiting affordability of glucometers—a challenge less prevalent in settings with universal healthcare coverage (52, 53). Similarly, the modest alcohol moderation rate (38%) diverges from Western studies but aligns with cultural norms in China, where alcohol is less emphasized in GDM guidelines (54–56). Notably, illiteracy’s outsized impact on practices (OR 88.7) exceeds magnitudes reported elsewhere, underscoring systemic inequities in China’s aging population (57–59).

Attitudinally, only 45% displayed positivity toward GDM management, lower than the 60% in Shang et al.’s (29) postpartum Chinese sample and the 72% in a Brazilian study (60). While 60% were open to diabetes education, only 36% viewed personal accountability as critical to GDM management. Younger women (20–30 years: OR 2.67) and those with primary education (OR 21.5) held more positive attitudes, whereas poor practices increased negative attitude odds (OR 5.56). Younger women (20–30 years; OR 2.67, 95% CI 1.94–3.69) and those with primary education (OR 21.5, 95% CI 13.0–36.1) showed more positive attitudes, consistent with evidence that early-life health optimism fosters engagement (61). Conversely, poor practices significantly increased the odds of a negative attitude (OR 5.56, 95% CI 4.48–6.91), suggesting a feedback loop, supported by Bandura’s self-efficacy theory (62–64). A family history of diabetes (OR 2.65, 95% CI 2.13–3.32) linked to positivity, possibly due to heightened awareness (64), while prior hospitalization predicted negativity (OR 0.55, 95% CI 0.41–0.74), reflecting psychological distress (65). Notably, a family history of diabetes conferred a two-and-a-half-fold greater likelihood of positive attitudes (adjusted OR 2.65, 95% CI 2.13–3.32). This observation aligns with social-cognitive models positing that vicarious experience and familial role-modeling enhance risk appraisal and outcome expectancy, thereby fostering adaptive disease perceptions. These findings suggest that harnessing family-centered counseling—particularly involving first-degree relatives with diabetes—may reinforce favorable attitudinal dispositions toward postpartum self-care.

The inverse relationship between poor practices and negative attitudes (OR 5.56) reflects cognitive dissonance: individuals with suboptimal practices may rationalize their behaviors through pessimistic attitudes (66, 67). Younger women’s positive attitudes may stem from greater exposure to digital health campaigns, which emphasize empowerment (68, 69). Conversely, the association between family history of diabetes and positive attitudes (OR 2.65) aligns with social cognitive theory, where familial experiences model proactive health behaviors (70, 85, 86).

Although our investigation centered on ante-partum KAP, these age-stratified patterns have critical postpartum ramifications. Women aged 51–60 years demonstrated the highest odds of poor practices (OR 12.7, 95% CI 8.79–18.4) despite relatively robust knowledge scores—an incongruence that may amplify metabolic risk after childbirth. Chinese cohort data indicate that > 50% of mothers with prior GDM develop type 2 diabetes within a decade, yet < 25% complete the recommended 6-to-12-week postpartum OGTT (16, 17). Older mothers frequently face entrenched lifestyle habits, multi-morbidity, and caregiving obligations that deprioritize follow-up. Our findings therefore advocate for age-tailored postpartum interventions combining structured glycemic surveillance, behavioral counseling, and digital reminders, aligned with Healthy China 2030 targets for non-communicable-disease control.

This study addresses critical gaps by providing a large-scale, comprehensive KAP assessment among Chinese women with GDM, a population underrepresented in global research despite China’s high prevalence (3). Unlike prior studies focusing narrowly on pregnant women (27), our inclusion of a broad age range and disease duration offers a lifecycle perspective, crucial for understanding long-term outcomes like T2DM risk. The identification of predictors such as illiteracy and unemployment extend prior work by quantifying socioeconomic barriers, informing targeted public health strategies (71, 72). Multi-level interventions—community education with visual aids, subsidized monitoring tools, and culturally tailored programs—can address illiteracy, income barriers, and accountability, while stress management could improve glycemic control. Given China’s 14.8% GDM prevalence, integrating education into prenatal care and enhancing rural healthcare infrastructure are critical policy steps.

The importance lies in its implications for China’s healthcare system amidst its epidemiological transition. With GDM linked to a sevenfold increased T2DM risk (73–75, 87), enhancing KAP is a priority to mitigate intergenerational metabolic disease burdens. Our findings highlight the need for culturally adapted interventions—e.g., addressing low awareness of eye exams (38%) and foot care (40%)—which are critical for preventing complications like retinopathy and neuropathy (76, 77). Furthermore, attitudinal barriers (e.g., 39% feeling misunderstood by providers) call for improved patient-provider communication, a known determinant of chronic disease outcomes (78, 79), aligning with WHO’s global diabetes strategy (80–82).

Strengths of this study include its large, demographically diverse sample of 3,426 Chinese women, which enhances statistical power and generalizability to urban populations. The rigorous application of multivariate logistic regression models identified critical sociodemographic and clinical predictors of gaps in KAP, such as illiteracy and income disparities, offering novel insights into the social determinants shaping GDM management. Additionally, this study is among the first to comprehensively assess bidirectional relationships between attitudes, practices, and knowledge in a low- and middle-income population in China, providing actionable evidence for designing culturally tailored, multi-level interventions to address GDM-related health inequities.

Although urban–rural disparities are evident, our data reveal that women with *secondary education* form an equally vulnerable cohort: they achieved moderate knowledge scores yet recorded the poorest attitude and practice indices. This pattern is emblematic of *fragmented health literacy*, which is sufficient to answer factual questions but insufficient to sustain complex self-management, particularly after childbirth when structured clinic contact diminishes. Targeted solutions must therefore move beyond conventional didactic sessions. Community health-worker outreach using visual aids (e.g., pictograms of postpartum OGTT timing and type 2 diabetes risk), narrative testimonials, and bi-directional mHealth messaging can convert partial awareness into postpartum action. Embedding these tools within existing maternal–child-health visits would widen reach in both urban and rural settings while minimizing literacy barriers.

This study has several limitations that warrant consideration. First, the cross-sectional design inherently limits causal inference and captures KAP only at a single antenatal timepoint. As such, we were unable to assess postpartum behavioral trajectories or longitudinal glycemic outcomes, which are essential to understanding the full continuum of GDM management. Second, the reliance on self-reported data may introduce recall and social desirability biases, particularly in rural settings where health workers facilitated paper-based data collection. Additionally, the diverse methods of data collection—digital surveys via WeChat in urban areas and paper forms in rural clinics—may have introduced measurement bias by disproportionately favoring tech-literate participants. Third, the 75% cut-off used for categorizing KAP status, although consistent with prior literature, may oversimplify the spectrum of behavioral engagement. The absence of objective clinical parameters (e.g., HbA1c or OGTT values) further limits the validation of self-reported practices. Future longitudinal work will incorporate objective biomarkers (HbA1c, postpartum OGTT) to validate self-reported practices. Finally, listwise deletion of missing responses (<2%) may have introduced minor bias, although its impact is likely negligible given the high overall response rate.

## Conclusion

This study highlights significant gaps in KAP related to GDM among Chinese women, reflecting a complex mix of educational, socioeconomic, and cultural barriers that undermine effective disease management. While many participants showed awareness of GDM’s complications, their limited grasp of comprehensive management strategies and inconsistent self-care practices reveal a critical disconnect that risks maternal and neonatal health. Stark disparities—linked to illiteracy, older age, and economic disadvantage—emphasize the need for targeted interventions to tackle these inequities. Additionally, low personal accountability and trust in healthcare providers point to deeper attitudinal challenges, requiring culturally sensitive approaches to boost empowerment and engagement. These findings urge the adoption of evidence-based, equity-driven strategies, such as accessible education, subsidized resources, and community-based initiatives, to curb GDM’s growing public health impact in China and its long-term effects. Future research should focus on longitudinal studies to test tailored interventions, explore digital health tools for improving KAP across diverse groups, and assess regional differences to guide a unified national policy. Priority should be given to women with secondary education, whose partial awareness often masks critical knowledge gaps; layered community outreach, culturally adapted visuals, and low literacy mHealth reminders in the postpartum window are vital to closing this equity gap and curbing long-term diabetes risk.

## Data Availability

The raw data supporting the conclusions of this article will be made available by the authors, without undue reservation.
